# Is There a Plastic Surgeon on Call? A Population-Based Cross-Sectional Study on Plastic Surgery Call Coverage in Ontario

**DOI:** 10.1177/22925503261479690

**Published:** 2026-09-01

**Authors:** Mira Madumere, Kimberley Yuen, Isabella Churchill, Farzana Akter, Yaqi Liu, Achilleas Thoma, Arthur Sweetman, Helene Retrouvey

**Affiliations:** 1 Michael G. DeGroote School of Medicine, 12362 McMaster University, Hamilton, ON, Canada; 2 Division of Plastic Surgery, Department of Surgery, 153004 McMaster University, Hamilton, ON, Canada; 3 Department of Surgery, 153004 McMaster University, Hamilton, ON, Canada; 4 Department of Economics, Health Policy PhD program, 248191 McMaster University, Hamilton, Canada; 5 Department of Health Research Methods, Evidence and Impact (HEI), 3710 McMaster University, Hamilton, ON, Canada

**Keywords:** call coverage, Ontario, emergent services, geographic distribution, plastic surgery, couverture de garde, Ontario, services d'urgence, répartition géographique, chirurgie plastique

## Abstract

**Introduction:**

Plastic surgery consultations are frequently required in emergency department settings; however, some hospitals lack plastic surgery call coverage. This study evaluated the geographic distribution of plastic surgeons providing call coverage within Ontario and the accessibility of emergency plastic services for the population.

**Methods:**

Demographic data on practicing plastic surgeons were obtained from the College of Physicians and Surgeons of Ontario database. Call coverage was determined by the number of surgeons by primary practice location and hospital affiliations per surgeon in each region. A survey was sent to switchboard personnel at all hospitals in Ontario to further assess call coverage. Results were reported as absolute counts and surgeons per 100 000 population—ie, population density ratios (PDRs).

**Results:**

A total of 289 plastic surgeons reported their primary practice location in Ontario. Most practice within Metropolitan Toronto and Central Ontario (66.8%). Northern Ontario had the lowest PDR compared to Metropolitan Toronto with the highest (PDR = 1.29 vs 3.72, 95% CI: 1.41-3.78, *P* < .001). For 254 hospitals identified, our survey response rate was 64%. Fifty-eight percent of responding hospitals reported no plastic surgery coverage. Over half of hospitals in Metropolitan Toronto and Central Ontario reported plastic surgery call coverage, compared to only 24% in Northern Ontario.

**Conclusions:**

This study demonstrates that 95 (58%) hospitals are without plastic surgery call coverage and highlights the discrepancies in the geographic spread of plastic surgeons in Ontario. Further studies are needed to determine the impact of this discrepancy on patient outcomes.

## Résumé

**Introduction:**

Les consultations en chirurgie plastique sont fréquentes aux services d’urgence, mais certains hôpitaux ne disposent pas de couverture de garde en chirurgie plastique. La présente étude évalue la répartition géographique des chirurgiens plasticiens qui assurent une couverture de garde en Ontario, ainsi que l’accessibilité de la population aux services de chirurgie plastique d’urgence.

**Méthodes:**

Les données démographiques sur les chirurgiens plasticiens en exercice proviennent de la base de données de l’Ordre des médecins et chirurgiens de l’Ontario. La couverture de garde a été déterminée selon le nombre de chirurgiens par lieu d’exercice principal et les affiliations hospitalières de chaque chirurgien dans les diverses régions. Les standardistes de tous les hôpitaux de l’Ontario ont reçu un sondage afin de mieux évaluer la couverture de garde. Les résultats ont été déclarés sous forme de nombres absolus et de chirurgiens sur 100 000 habitants, qui correspondent aux rapports de densité de population (RDP).

**Résultats:**

Au total, 289 chirurgiens plasticiens ont déclaré leur lieu principal de pratique se situait en Ontario. La majorité exerce dans le Grand Toronto et le Centre de l’Ontario (66,8 %). Le Nord de l’Ontario présentait le RDP le plus faible et le Grand Toronto, le plus élevé (RDP = 1,29 par rapport à 3,72, IC à 95 % : 1,41–3,78, p<0,001). Le taux de réponse au sondage dans les 254 hôpitaux retenus s’établissait à 64 %. Ainsi, 58 % des hôpitaux répondants ont déclaré ne pas disposer de couverture de garde en chirurgie plastique. Plus de la moitié des hôpitaux du Grand Toronto et du Centre de l’Ontario ont indiqué en posséder une, par rapport à seulement 24 % de ceux du Nord de l’Ontario.

**Conclusions:**

La présente étude démontre que 95 hôpitaux (58 %) ne disposent d’aucune couverture de garde en chirurgie plastique et fait ressortir les écarts dans la répartition géographique des chirurgiens plasticiens de l’Ontario. D’autres études devront être réalisées pour déterminer les conséquences de ce phénomène sur le pronostic des patients.

## Introduction

Plastic surgeons are vital in the management of upper extremity injuries, craniofacial trauma, burns, and soft tissue infections, which often present first to the emergency department (ED).^1^ In fact, hand injuries alone account for up to 28.6% of all cases seen within an ED.^2–3^ Emergency presentations that require the aid of a plastic surgeon can be time-sensitive, especially when limb- or life-threatening.^4–7^ Thus, consistent access to a plastic surgeon is necessary for timely surgical assessment, optimal patient management, and equitable patient access to care.^8,9^

Despite this necessity, access to on-call plastic surgery services remains an ongoing issue in Canada.^10,11^ On average, the waitlist time for an urgent plastic surgery consultation across the country is 11 days.^10^ Not only is plastic surgery coverage lacking in Canada, but the distribution of plastic surgeons is skewed toward major urban centers such as Toronto, Vancouver, or Montreal.^10^ A mere 2% of plastic surgeons in Canada reported working in a rural community, which is concerning, as the burden of plastic surgery-related injuries such as burns, craniofacial, and hand trauma is higher, and often more severe in rural areas.^11–13^ Without sufficient plastic surgery call coverage support, many patients in rural settings are left vulnerable to delays in treatment due to inaccessible care, or may face travel burdens to receive necessary care.^8,14,15^

Ontario is Canada's most populated province, with almost 16.2 million inhabitants; it is serviced by 275 plastic surgeons—the highest number within the country.^11,16,17^ While the literature states the vast majority of plastic surgeons practice within large city centers in Ontario, no studies to date have examined how this relates to on-call plastic surgery services.^18^ Therefore, the aim of this study was to characterize the geographic spread of plastic surgery call coverage within Ontario, and to evaluate current gaps in delivery of plastic surgery services. Understanding these gaps will enable advocacy for interventions that enhance equitable access to plastic surgery care for Ontarians.

## Methods

### Study Design and Population

We conducted a population-based cross-sectional study on the call coverage of plastic surgeons within Ontario. The College of Physicians and Surgeons of Ontario (CPSO) website was utilized to identify plastic surgeons in Ontario. The inclusion criteria were physicians within the CPSO with a Royal College of Physicians and Surgeons of Canada (RCPSC) specialist certification in plastic surgery and physicians with an independent licensing certificate. Physicians without an independent licensing certificate and those completing post-graduate medical training were excluded. The list of hospitals within Ontario was obtained from the Government of Ontario website,^19^ and a survey was administered to each hospital site to characterize their access to plastic surgery consulting services.

### Survey Design and Administration

The survey was created de novo from literature review and discussion among the authors of this study.^8,20^ The survey contained 8 questions addressing the accessibility of on-call coverage, the delivery method of call (in person or telemedicine), and management plans at hospital centers where plastic surgery on-call coverage was absent. Elements of the survey quantified plastic surgeons who provide call coverage, call obligation of each surgeon (mandatory number of call shifts per month), the structure and variability of plastic surgery call services, and call deficits at each hospital (days without plastic surgery call coverage within the last month).

The survey was administered to switchboard personnel at each hospital site within Ontario via telephone from January to June 2025. If switchboard personnel were unable to answer the survey questions, an electronic copy of the survey was sent to individuals via email who were identified by switchboard staff as having knowledge of plastic surgery on-call coverage. Reminder emails were sent 1 week later to non-responders. Individuals were given a total of 2 weeks to respond to the survey. Survey data were recorded in Microsoft Excel. A copy of the survey can be found in the Online Appendix.

### Outcomes and Outcome Measures

**Figure 1.**
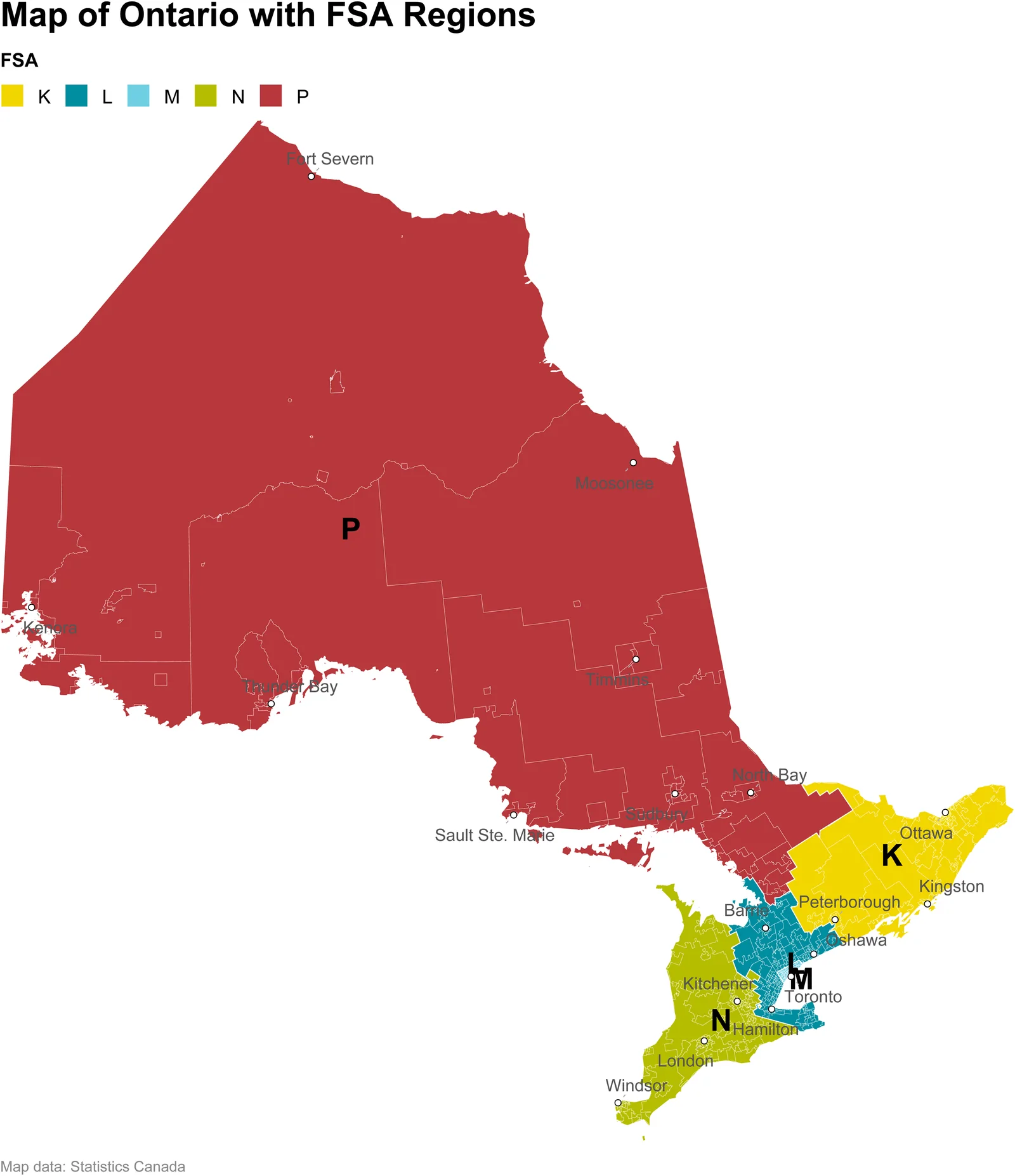
Geographic regions within Ontario based on FSA codes (postal districts). Regions included Northern Ontario (P), Southwestern Ontario (N), Central Ontario (L), Metropolitan Toronto (M), and Eastern Ontario (K).

The primary outcome of this study was defined as the geographic distribution of plastic surgery call coverage across Ontario. Our primary outcome was measured by the number of plastic surgeons per geographic region. The geographic region was represented by Forward Sortation Area (FSA), which represents the first 3 digits of a postal code and the first digit of an FSA corresponds to a postal district in Canada **(**Figure 1**)**.^21^ Plastic surgery coverage was calculated in 2 ways: (1) number of plastic surgeons per geographic region based on primary practice location (ie, 1 plastic surgeon per location), and (2) number of affiliations per plastic surgeon (ie, if a plastic surgeon had 3 affiliated hospitals, then each affiliated location counted as having a surgeon providing coverage). Hospital privilege and hospital affiliation were used interchangeably and represent a hospital location with an associated practicing plastic surgeon.

Secondary outcomes included the comparison of such distribution to the population distribution within Ontario and to outline the geographic spread of hospitals with and without plastic surgery consultation services in Ontario. These outcomes were measured by the density of plastic surgeons per geographic region and 100 000 population, as well as the number of hospital sites with and without access to a plastic surgeon on call in each geographic region.

### Data Collection

Demographic data on all Ontario plastic surgeons were extracted from the CPSO website in January 2025. To avoid restrictions on search results that exceed 100, the search was conducted by each city in Ontario. For cities that displayed more than 100 surgeons and thus were restricted in results, the search was conducted by the first 2 FSA digits. Demographic data extracted included the name of the plastic surgeon, location(s) of practice and hospital privileges, number of hospital privilege locations, and year of RCPSC certification.

### Statistical Analysis

Descriptive statistics were used to summarize demographic and survey data. Geographic data were organized according to postal code. Results were reported as absolute counts, population density ratios (PDRs) represented as the ratio of plastic surgeons per 100 000 population in each geographic region, means with standard deviations, and medians with interquartile ranges (IQR), as appropriate. Population estimates were obtained from the 2021 Statistics Canada census data. Geographic maps were generated to illustrate the postal districts in Ontario, the distribution of plastic surgeons across the province, as well as the spread of hospitals with and without access to plastic surgery services. A generalized linear model (GLM) with a Poisson distribution and log link function was used to assess differences in the number of plastic surgeons across Ontario's 5 geographic regions (Eastern (K), Central (L), Metropolitan Toronto (M), Southwestern (N), and Northern (P)). The natural logarithm of the population for each region was included as an offset term to account for population size, effectively modeling the rate of surgeons per population.

## Results

**Figure 2:**
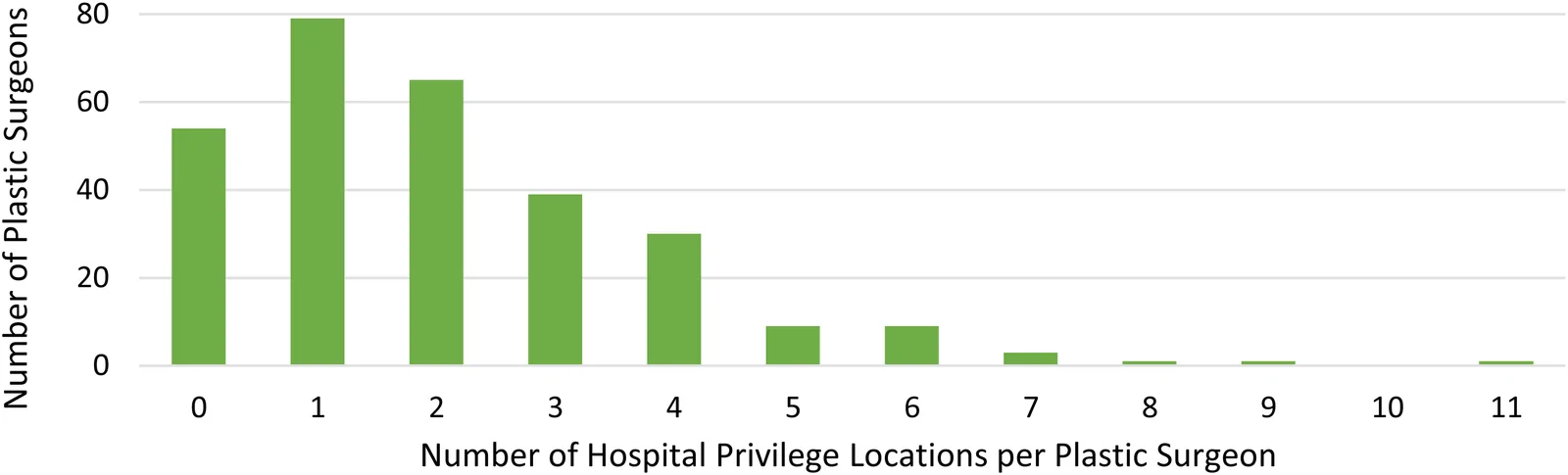
: Number of hospital privilege locations per plastic surgeon in Ontario. Hospital privilege location data were obtained from the CPSO database.

From the CPSO website, 291 plastic surgeons were identified. Two surgeons did not have a primary practice listed in Ontario, 1 of whom did not have a listed practice location in Ontario. This left 290 plastic surgeons with a listed practice location in Ontario and 289 with a primary practice location in Ontario. In each geographic region in Ontario, there are at least twice as many male plastic surgeons as there are female surgeons. Year of RCPSC certification ranged from 1972 to 2024; the median year of certification was 2011. The median number of hospital privileges per plastic surgeon was 2 and ranged from 0 to 11, and 79 (27.2%) of plastic surgeons within Ontario had only 1 affiliated site **(**Figure 2**).** Most surgeons (*n* = 183, 63.1%) had between 1 and 3 hospital affiliation sites. Demographic and distribution data can be seen in Table 1.

**Table 1.** Demographic Data of Plastic Surgeons Practicing in Ontario.

Plastic Surgeons Practicing in Ontario	*N*				
CSPO Registrants	290				
Total number of listed primary practice locations	289/290				
Total number of hospital affiliations of all plastic surgeons in Ontario	363/290				

	Eastern Ontario (K)	Central Ontario (L)	Metropolitan Toronto (M)	Southwestern Ontario (N)	Northern Ontario (P)
Population	2 196 966	5 599 314	2 794 323	2 779 475	853 863
Number of plastic surgeons (N (%), PDR) based on primary practice location (total *N* = 289)	43 (14.9%), 1.96	89 (30.8%), 1.59	104 (36.0%), 3.72	42 (14.5%), 1.51	11 (3.8%), 1.29
Number of hospital affiliations (*N* (%), PDR) (total *N* = 290)	46 (15.9%), 2.09	114 (39.3%), 2.04	136 (46.9%), 4.87	49 (16.9%), 1.76	18 (6.2%), 2.11
Gender					
Man	32	56	72	26	7
Woman	11	33	32	16	4
Language					
English	43	89	104	42	11
French	14	9	12	5	1
					
Afrikaans			1		1
Arabic		3	2	1	
Armenian		1			
Chinese Dialects	1	3	6		
Danish			1		
Dutch		1	1	1	1
Farsi		1	2	1	
German		1	4	1	
Greek	2	1	1	1	
Hebrew		1	4	1	
Hindi	1		3		1
Hungarian			1		
Indonesian				1	
Italian		4	1	1	
Macedonian		1			
Marathi	1				
Persian	1				
Polish		3	1		
Portuguese		1			
Punjabi/Panjabi			2		
Romanian	1				
Russian			1		
Spanish			1	1	
Ukrainian			2		
Urdu			2		

### Geographic Distribution of Plastic Surgeons With Hospital Privileges

**Figure 3.**
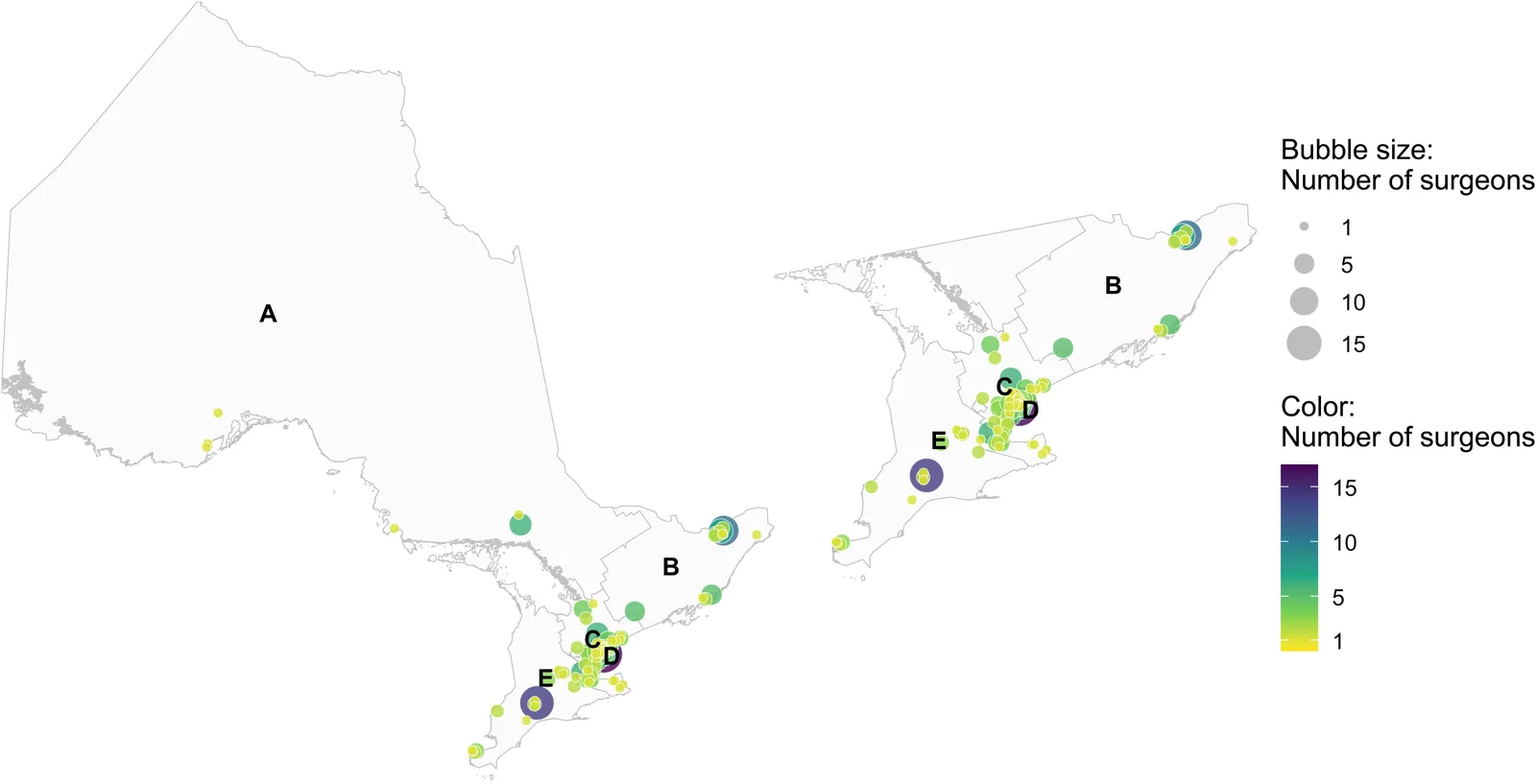
Primary practice locations of plastic surgeons in Ontario. Regions included Northern Ontario (A), Eastern Ontario (B), Central Ontario (C), Metropolitan Toronto (D), and Southwestern Ontario (E). Primary practice location data were extracted from the CPSO database.

**Figure 4.**
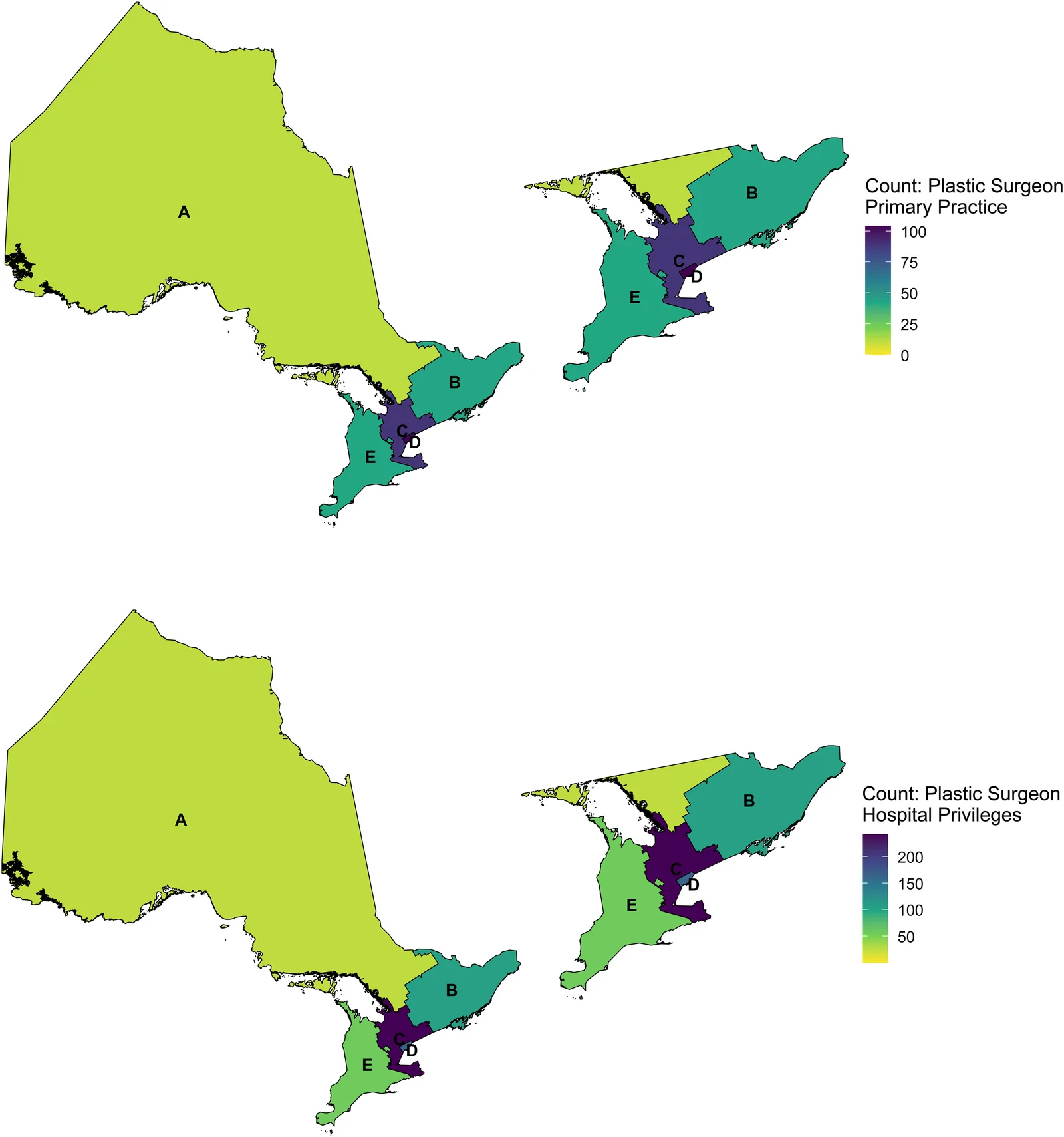
(A) Number of plastic surgeons across various geographic regions in Ontario based on primary practice location (1 postal code per surgeon). Regions included Northern Ontario (A), Eastern Ontario (B), Central Ontario (C), Metropolitan Toronto (D), and Southwestern Ontario (E). Hospital privilege locations were extracted from the CPSO database. (B) Number of plastic surgeons across various geographic regions in Ontario based on all hospital privileges (each plastic surgeon could have up to 11 postal codes). Regions included Northern Ontario (A), Eastern Ontario (B), Central Ontario (C), Metropolitan Toronto (D), and Southwestern Ontario (E). Hospital privilege locations were extracted from the CPSO database.

According to primary practice location, most plastic surgeons in Ontario practice within Metropolitan Toronto and Central Ontario (66.8%), with even more surgeons with a hospital affiliation within these regions (86.2%). Notably, Metropolitan Toronto accounts for almost half (46.9%) of all hospital affiliations in Ontario. Southwestern and Eastern Ontario had similar numbers of plastic surgeons (*n* = 42, 14.5% and *n* = 43, 14.9%, respectively), followed by Northern Ontario with the fewest (*n* = 11, 3.8%). Findings were similar when calculated using all hospital affiliations, except for Northern Ontario, whose PDR almost doubled from 1.29 to 2.11, leaving Southwestern Ontario with the lowest PDR of 1.76 (Table 1). The distribution of plastic surgeons based on primary practice location and total number of hospital privileges across different postal districts in Ontario can be seen in Figure 3 and 4**.**

### Plastic Surgeons and the Population They Serve: Population Density Ratio (PDR)

**Figure 5.**
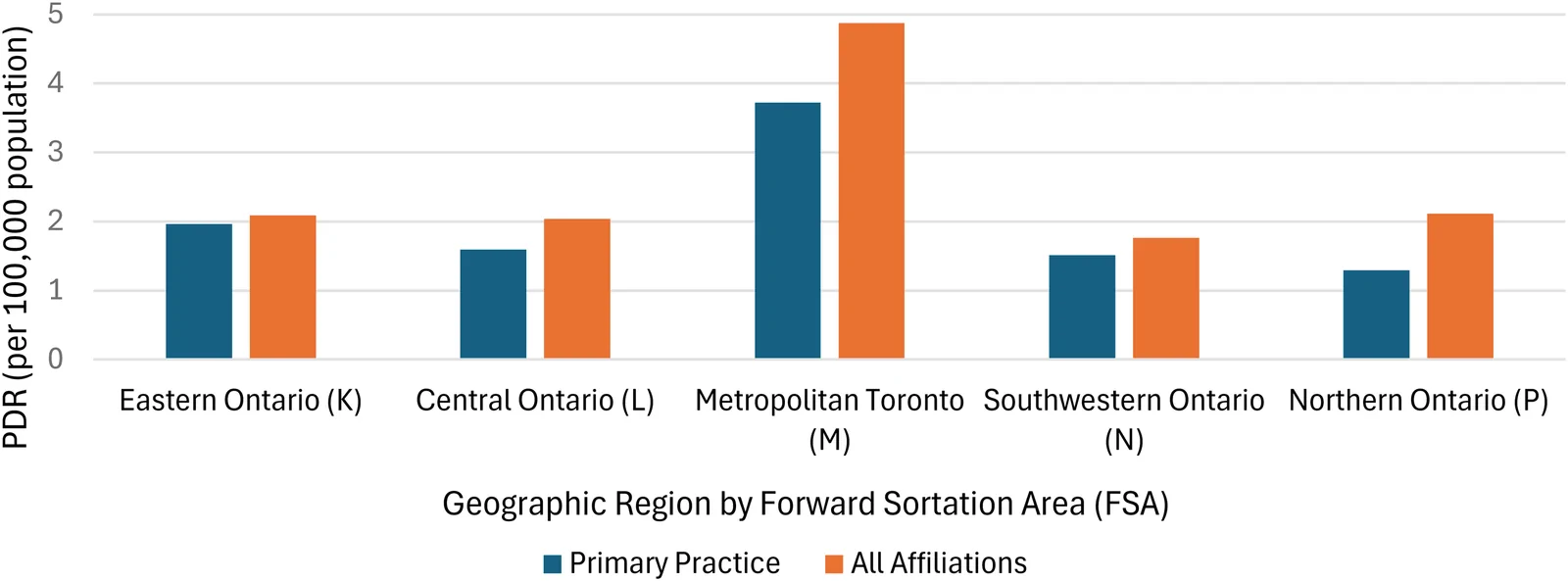
Comparison of population density ratio (PDR) of plastic surgeon to 100 000 population between primary practice and all hospital affiliations across various geographic regions in Ontario. Plastic surgeon and population data were acquired from CPSO and Statistics Canada, respectively.

Using primary practice location, Northern Ontario had the lowest ratio of plastic surgeons to 100 000 population density, with a PDR of 1.29. Metropolitan Toronto, at 3.72, was found to have a PDR more than double that of almost all other regions. The PDR values for Southwestern Ontario, Central Ontario, and Eastern Ontario were more comparable (1.51, 1.59, 1.96, respectively). The comparison of PDR across postal districts based on primary practice location and number of hospital affiliations can be seen in Figure 5. The generalized linear model with Poisson distribution and log link demonstrated good fit (AIC = 39.47) and a significant overall effect of region, *χ*^2^(4) = 62.75, *P* < .001 (Table 2). After adjusting for population, Metropolitan Toronto had a significantly higher density of plastic surgeons compared to Northern Ontario (Exp(B) = 2.31, 95% CI: 1.41-3.78, *P* < .001). No statistically significant differences were observed for Eastern, Central, or Southwestern Ontario.

**Table 2.** Results From the Generalized Linear Model (GLM) With Poisson Distribution and log Link to Evaluate the Association Between Postal Districts Across Ontario and the Number of Plastic Surgeons.

Region	B	SE	Exp(B)	95% CI for Exp(B)	*P*
(Intercept)	−10.767	0.236	—	—	<.001
Eastern Ontario (K)	−0.007	0.278	0.993	[0.576, 1.713]	.981
Central Ontario (L)	−0.035	0.254	0.966	[0.587, 1.588]	.891
Metropolitan Toronto (M)	0.837	0.251	2.309	[1.412, 3.775]	<.001
Southwestern Ontario (N)	−0.179	0.276	0.836	[0.487, 1.435]	.517
Northern Ontario (P, reference)	0	—	1.00	—	—

### Survey Responses on the Availability of Plastic Surgery Services, Call Structure, and Comparison of Hospitals With and Without Call Coverage by Postal District in Ontario

A total of 254 hospital locations were identified in Ontario. Our survey was answered by switchboard personnel at 163 hospitals, giving a response rate of 64%. All survey respondents were acquired via telephone interviews. Of the 163 surveyed hospitals, 58% reported they did not have access to plastic surgery consulting services. However, these hospitals had the means to transfer a patient to a hospital site with a plastic surgeon if required. Only 68 (42%) hospitals had access to plastic surgery consulting services. However, within this number, most have access to an on-site plastic surgeon if needed (*n* = 61, 90%). Regarding call coverage providers, 32 of 33 (96.4%) reported having between 1 and 20 plastic surgeons who can provide call coverage, and 9 of 61 responses (14.7%) reported their plastic surgeons take between 1 and 7 days of call in a month. Forty-six of 54 hospitals (85%) reported having daily call coverage in the past month. The structure of call shifts for plastic surgery mainly tended to be scheduled as 24-h shifts (*n* = 42, 71.1%). Complete survey responses regarding the details of call coverage can be seen in Table 3.

**Table 3.** Survey Responses From Switchboard Personnel or Next Most Responsible Individual on Plastic Surgery Call Coverage Details.

	*N* (%)
*Access to Plastic Surgery*	*N* = 163
Yes	68 (42)
No	95 (58)
*Access to on-Site Consultations*	*N* = 63
Yes	61 (96.8)
I do not know	2 (3.2)
*Number of Plastic Surgeons who Provide Call Coverage*	*N* = 33
Between 1 and 5	21 (65.6)
Between 6 and 10	5 (15.6)
Greater than 10	6 (18.8)
I do not know	1 (3.0)
*Number of Mandatory Days per Month of Call per Staff Surgeon*	*N* = 61
Zero days	0 (0.0)
Between 1 and 5 days	5 (55.0)
Between 6 and 10 days	4 (45.0)
Greater than 10 days	0 (0.0)
I do not know	52 (85.2)
*Number of Days without Plastic Surgery Call Coverage Within the Last Month*	*N* = 53
0 (Never)	46 (86.8)
1 to 5 (Rarely)	1 (1.9)
5 to 10 (Occasionally)	2 (3.8)
10 to 15 (Frequently)	0 (0.0)
Greater than 15 days (Very Frequently)	4 (7.5)
*Structure of Call Coverage*	*N* = 59
24-h shift	42 (87.5)
12-h shift	4 (8.3)
8-h shift	1 (2.1)
No defined structure	1 (2.1)
I do not know	11 (18.6)
*Variance of Call Coverage Structure on Holidays and/or weekends*	*N* = 57
Yes	20 (42.0)
No	29 (58.0)
I do not know	8 (14.0)
*Ability to Transfer Patients to a Center with Access to Plastic Surgery Consulting Services*	*N* = 83
Yes	78 (100.0)
I do not know	5 (6.0)

**Figure 6.**
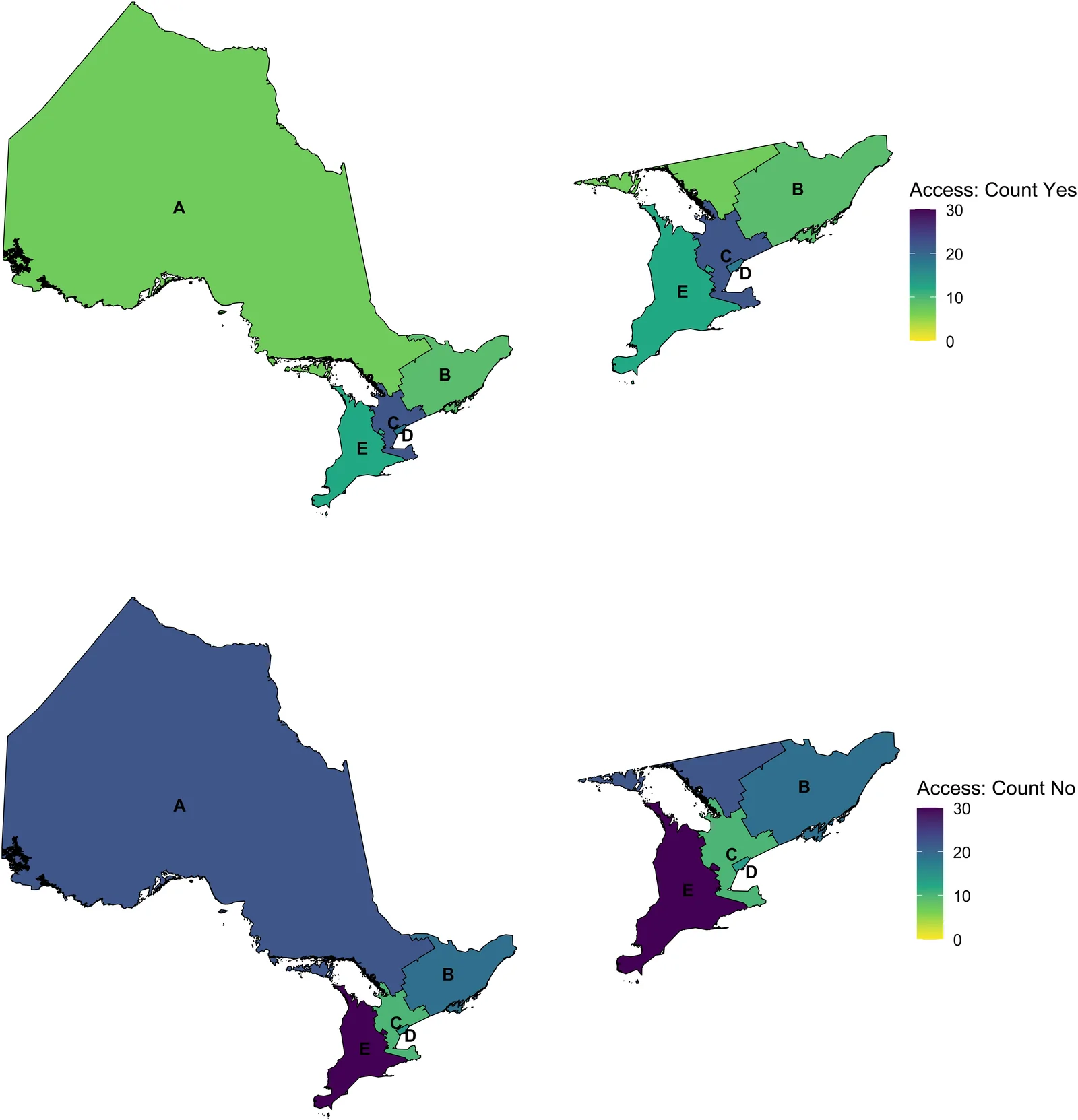
Proportion of hospitals in Ontario (A) with access to plastic surgery call coverage and (B) without access to plastic surgery call coverage: Northern Ontario (A), Eastern Ontario (B), Central Ontario (C), Metropolitan Toronto (D), and Southwestern Ontario (E).

When stratified by geographic region, Northern Ontario, Southwestern Ontario, and Eastern Ontario had more hospitals *without* plastic surgery coverage than *with* it. The proportion of hospitals without coverage was almost 3 times higher within Northern Ontario, 2.5 times within Southwestern Ontario, and 2 times within Eastern Ontario compared to hospitals without coverage. Southwestern Ontario had the most responses (*n* = 42) and the highest number of hospitals without plastic surgery coverage (*n* = 30, 71%). Northern Ontario had the highest proportion of hospitals without plastic surgery coverage (*n* = 22, 76%). Meanwhile, Central Ontario and Metropolitan Toronto both had 32 responses each. Of the 32 responses each, 56% of hospitals in Metropolitan Toronto and 69% of hospitals in Central Ontario had plastic surgery coverage. However, for both regions, one third to one half of hospital respondents still did not have plastic surgery coverage. These findings are represented in Figure 6 and Table 4.

**Table 4.** Hospital Access to Plastic Surgery Call Coverage by Postal District in Ontario.

Geographic Region	Total *N* (%)	Hospitals with Plastic Surgery Coverage *N* (%)	Hospitals Without Plastic Surgery Coverage *N* (%)
Northern Ontario (P)	29(100)	7(24)	22 (76)
Southwestern Ontario (N)	42 (100)	12(29)	30 (71)
Central Ontario (L)	32(100)	22(69)	10 (31)
Metropolitan Toronto (M)	32(100)	18(56)	14 (44)
Eastern Ontario (K)	28(100)	9 (32)	19 (68)

## Discussion

We sought to identify the geographic distribution of plastic surgery call coverage across Ontario. Our findings demonstrated disproportionate representation of plastic surgeons, with higher concentrations in more urban centers, and limited availability in rural and northern regions. While 56% of hospitals in Metropolitan Toronto have access to a surgeon on call, 3 in 4 hospitals in Northern Ontario do not. This imbalance raises concerns for equitable access to emergency plastic surgery services, timely patient care, and the sustainability of on-call workloads for plastic surgeons in underserviced areas.

The demand for plastic surgeons has been assessed in previous studies. In Canada, the expected ratio of plastic surgeons per 100 000 people was 1.55 based on findings from the Canadian Plastic Surgery Workforce Analysis in 2017.^11^ Our study found disparities in the ratio of plastic surgeons to population across different geographic regions. Specifically, Northern Ontario and Southwestern Ontario did not meet the expected ratio, with PDRs of 1.29 and 1.51, respectively. Moreover, PDRs for Central are not dissimilar to those of Southwestern and Northern Ontario. These comparable ratios suggest that surgeon population alone does not capture barriers to care. One such barrier includes travel burden due to geographic spread. Central and Southwestern Ontario have populations 3 to 7 times larger than Northern Ontario, with more hospitals and surgeons distributed across multiple sites.^22^ In contrast, Northern Ontario has fewer hospitals with on-call plastic surgeons, and most residents live more than 30 min from their nearest hospital, irrespective of travel method.^23^ For time-sensitive trauma and hand injuries, these travel barriers increase the risk of poorer outcomes. Our results are similar to those of Teng et al,^24^ who found rural and suburban areas in the United States tend to lack adequate access to plastic surgery services. The disproportionate distribution of healthcare in rural compared to urban settings has been associated with higher mortality rates and hospital costs.^25^ Improving the delivery of plastic surgery in remote regions, then, could lead to improvements in health outcomes and healthcare costs across the nation.

The clustering of plastic surgeons within urbanized centers like Metropolitan Toronto could be explained by the growing interest in cosmetic-based practice. In 2024, Hunt et al^26^ investigated factors that impacted practice decisions in early-career plastic surgeons and residents in Canada. They found most early-career plastic surgeons (77%) and residents (73%) desired to have a mixed aesthetics-reconstructive practice.^26^ This is a sizable increase from a previously reported 24%.^11^ The appeal of a cosmetic practice may draw plastic surgeons toward larger, more urbanized centers, leaving rural communities with inadequate access to plastic surgery care. Evidently, fewer plastic surgeons are servicing rural communities. Almost 20 years ago, the Canadian plastic surgery workforce survey showed 12% of plastic surgeons worked in a rural setting, which decreased to 2% in the 2018 survey.^10,11^

Two persistent issues continue to contribute to the lack of plastic surgery coverage in Canada: (1) increase in aesthetic practices and (2) decrease/low numbers of plastic surgeons practicing in rural areas. An editorial by Dr Achilleas Thoma^27^ published in *Plastic Surgery* earlier this year highlighted these exact issues. Dr Thoma proposed that plastic surgery trainees should participate in a “return to service” type agreement, by serving their community for a minimum time period (eg, 10 years) to obtain the society's membership, before embarking on a solely aesthetic practice. Recent graduates should be assigned to regional hospitals based on need, and a licence to practice should be coupled with such employment. Nationwide commitment is required to train future plastic surgeons to meet the needs of our country.

Implementation of certain strategies could address the issue of call coverage. For instance, rural rotations during training could be mandated to provide clinical and contextual exposure. In the United States, other specialties have found that such rotations improved the number of practicing specialists in rural communities.^28–30^ Incentivization of rural experiences is already underway for medical trainees in Ontario. The Northern Ontario Resident Streamlined Training and Reimbursement Program (NORSTAR) provides subsidized learning opportunities for residents and fellows interested in supporting communities in Northern Ontario.^31^ On an international level, this has proven to be effective within rural communities in Portugal. In 2015, the Government of Portugal launched an incentive package for physicians to practice in underserved areas, which included a rural practice for 5 years and financial incentives (bonus over 5 years, vacation days).^32^ As a result, Portugal experienced a 14-fold increase in physicians practicing within rural areas.^32^ Understanding the needs of plastic surgeons and implementing incentives to attract them to underserviced areas in Ontario could be an effective way of providing equitable access to plastic surgery-related healthcare, while improving the work environment of plastic surgeons practicing in these regions.

### Limitations

The findings of our study should be interpreted in the context of its limitations. We used the CPSO website as a barometer for active plastic surgeons based on their (1) primary practice location and (2) all affiliated hospital locations. Primary practice location may underestimate service to an area, as surgeons with multiple hospital affiliations could cover many areas, while counting all affiliated hospital locations as if each represented access to a plastic surgeon may overestimate coverage. Moreover, the data on primary practice location does not exclude surgeons with cosmetic practices who may have no hospital affiliation. Utilizing CPSO data, however, was the only way to acquire comprehensive data on plastic surgeons in Ontario, as accreditation from the organization is mandatory. Additionally, our study utilized population data from the 2021 Canadian census. The increase in Ontario's population between 2021 and the year this study was conducted (2025)^17^ could have led to an overestimation of our population density ratios. Nevertheless, the 2021 census provided the most recent data for the postal code-level analysis vital to our study. Lastly, hospital personnel (ie, switchboard) who completed our survey could have inaccurate knowledge of a given hospital's plastic surgery on-call system. Despite this, the information provided was the most accurate given this limitation, as there were no other means of obtaining knowledge regarding a hospital's call schedule.

## Conclusion

There is an uneven distribution of plastic surgery on-call coverage within Ontario. Namely, the majority of both the number of plastic surgeons and the number of hospitals with access to plastic surgery on-call services are greater within Metropolitan Toronto, despite the region's small size. Individuals in Northern Ontario have far less access to a plastic surgeon on call. This study should prompt the development of strategies that enhance patient access to care in rural areas. Furthermore, future studies should investigate the number of licensed plastic surgeons in Ontario who participate in call coverage.

## Supplemental Material

sj-docx-1-psg-10.1177_22925503261479690 - Supplemental material for Is There a Plastic Surgeon on Call? A Population-Based Cross-Sectional Study on Plastic Surgery Call Coverage in Ontario Supplemental material, sj-docx-1-psg-10.1177_22925503261479690 for Is There a Plastic Surgeon on Call? A Population-Based Cross-Sectional Study on Plastic Surgery Call Coverage in Ontario by Mira Madumere, Kimberley Yuen, Isabella Churchill, Farzana Akter, Yaqi Liu, Achilleas Thoma, Arthur Sweetman and Helene Retrouvey in Plastic Surgery
