# Supplementary material for: Is There a Plastic Surgeon on Call? A Population-Based Cross-Sectional Study on Plastic Surgery Call Coverage in Ontario
Source: Plast Surg (Oakv). 2026 Sep 1:22925503261479690. Online ahead of print. doi: 10.1177/22925503261479690 (PMC13534362; doi:10.1177/22925503261479690)
Supplement: Supplementary file 1 [file sj-docx-1-psg-10.1177_22925503261479690.docx]

**Appendix**

1. Does your hospital have access to plastic surgery consulting services?
   1. Yes
   2. No
2. If yes, is the consultant available on site to see the patient?
   1. Yes, if needed
   2. No- consultant only available via phone
   3. Other-
3. How many plastic surgeons provide plastic surgery call coverage at your hospital?
   1. [short answer response]
4. How many days of call does each staff plastic surgeon have to cover per month?
   1. [short answer response]
5. Within the last month, on average, how many days did your hospital lack plastic surgery call coverage?
   1. 0 (Never)
   2. 1-5 (Rarely)
   3. 5-10 (Occasionally)
   4. 10-15(Frequently)
   5. more than 15 days (Very Frequently)
6. How is call coverage for plastic surgery structured at your hospital?
   1. 24-hour call coverage
   2. Daytime shift (ex. 8, 12 hours long)
   3. Nighttime shift (ex. 8, 12 hours long)
   4. Other
7. Does the structure of plastic surgery coverage differ during different days of the week, weekends, or holidays?
   1. [short answer: ex. Yes- no call on weekends]
8. If you DO NOT have an affiliated plastic surgery service in your hospital, are patients with plastic surgery-related concerns directed to another hospital via direct transfer?
   1. Yes
   2. No (explain)
   3. I do not know
   4. Other comments
